# The stability of pain phenotypes in people with hand osteoarthritis – results from the NOR-HAND study

**DOI:** 10.1016/j.ocarto.2026.100745

**Published:** 2026-01-14

**Authors:** Daniel H. Bordvik, Elisabeth Mulrooney, Pernille Steen Pettersen, Marthe Gløersen, Lene Maria Sundbakk, Tuhina Neogi, Ingvild Kjeken, Ida K. Haugen

**Affiliations:** a Center for Treatment of Rheumatic and Musculoskeletal Diseases (REMEDY), Diakonhjemmet Hospital, Oslo, Norway; b Oslo Metropolitan University, OsloMET, Faculty of Health Sciences, Institute of Rehabilitation Sciences and Health Technologies, Oslo, Norway; c Rehabilitation West and the Norwegian Women's Public Health Association Haugesund, Haugesund, Norway; d Section of Rheumatology, Boston University Chobanian & Avedisian School of Medicine, USA; e Faculty of Medicine, University of Oslo, Norway

**Keywords:** Pain phenotyping, Hand osteoarthritis, Latent transition analyses

## Abstract

**Objective:**

In a hand osteoarthritis (OA) cohort, we aimed to explore pain phenotypes and characterize their stabilities using the multidimensional framework developed by The Initiative on Methods, Measurement and Pain Assessment in Clinical Trials (IMMPACT).

**Methods:**

We included participants attending the visits at baseline (2016–17) and follow-up (2019–21) in the Nor-Hand study. To identify pain phenotypes (i.e., classes), we conducted latent transition analyses modelling self-reported pain severity, neuropathic-like pain, fatigue, sleep, anxiodepressive symptoms, pain catastrophizing, and quantitative sensory testing (QST). We compared a model including QST with a model without. Longitudinal stability of classes was assessed. Changes in indicator variables across participants with vs. without between-class transitioning were compared.

**Results:**

We analysed 213 participants (86.9 % women) with a baseline mean age (standard deviation) of 60.9 (6.0) years. Both models identified four classes exhibiting similar results on differences in pain severity and psychosocial burden (kappa 0.91). Although phenotype stability varied (probability range: 0.48–0.95), most participants (_∼_80 %) remained in the same class at both visits. Most participants transitioning between classes shifted to a less severe pain class (32/44 (72.7 %) in the QST model; 36/45 (80.0 %) in the clinical model), showing larger improvements in pain and psychosocial burden than non-transitioners.

**Conclusion:**

Four distinct pain phenotypes were identified among persons with hand OA. Excluding QST from our model did not influence phenotype composition or characteristics. Various longitudinal phenotype stability was observed. Between-class transitions were often characterized by less pain and psychosocial burden, potentially due to regression to the mean or improved disease coping.

## 1 Background

The heterogeneity of pain across patients with hand osteoarthritis (OA) is still poorly understood [1,2]. Pain experience in hand OA is composed by an intricate, multi-level interplay extending beyond the joints, influenced by biological and psychosocial factors such as the central nervous system, affective states, and cognitive aspects [[2], [3], [4], [5], [6]]. Research is needed to identify pain mechanisms in hand OA subgroups to develop tailored strategies beyond the “one-size-fits-all” approach [1,2,7].

Pain phenotyping stratifies heterogeneous populations into subgroups based on traits or characteristics influencing the pain experiences [8]. It has gained attention as a useful tool to better understand pain, particularly within knee OA [7]. However, most studies have been cross-sectional, and there is limited knowledge regarding the stability of phenotypes over time [7,8]. In persons at risk of knee OA, changes of pain phenotype membership over time were found, although most patients remained in their baseline class [9]. While pain phenotypes and their six-year stability in persons with various hand conditions have been documented, data specific to hand OA are warranted [8,10].

Comparisons of pain phenotypes across previous OA studies are challenged by differing clustering methods and selection of variables [7]. Also, the biopsychosocial nature of pain has not been sufficiently mirrored in the selection of characteristics in some studies [7,[11], [12], [13]].

To overcome this, an initial framework established by the Initiative on Methods, Measurement, and Pain Assessment in Clinical Trials (IMMPACT) group, was recently recommended being the benchmark for classifying pain phenotypes in forthcoming OA research [11,14]. Based on their phenotype definition, a set of patient-reported characteristics and symptoms as well as quantitative sensory testing (QST) of pain sensitization is recommended. QST includes the use of specific instruments and methodologies for neurophysical assessment of pain response to stimuli [14]. Interestingly, sensitivity analyses from one study revealed that statistical models using only patient-reported IMMPACT variables effectively identified pain phenotypes cross-sectionally in advanced knee OA. These models performed comparably to main models with QST measures like pain threshold tests and conditioned pain modulation [11]. The extent to which QST is needed for adequacy of pain phenotyping in hand OA needs to be understood. No previous studies have used the IMMPACT framework to explore pain phenotypes or their stabilities in the context of hand OA.

Guided by the IMMPACT framework, we aimed to explore the characteristics and stability of pain phenotypes using self-reported indicators alone and combined with QST in a longitudinal study of persons with hand OA [14]. In addition, we aimed to compare demographic and clinical characteristics in persons transitioning between phenotypes compared with those remaining stable.

## 2 Material and methods

### 2.1 Study design and population

The Nor-Hand study is a cohort of persons with hand OA recruited from the outpatient rheumatology clinic, or a multidisciplinary OA course at Diakonhjemmet Hospital, Oslo, Norway (https://clinicaltrials.gov, NCT03083548). We utilized data from participants attending both the baseline examination in 2016–17 and the follow-up visit in 2019–21. Protocols describing the inclusion and exclusion criteria and data collections at both timepoints have been published [15,16]. At baseline, we included participants aged 40–70 years with OA in at least one thumb base or finger joint verified by ultrasound and/or clinical examination performed by a rheumatologist. At both visits, people with systemic inflammatory joint disease or psoriasis were excluded. Written informed consent was obtained from all participants prior to inclusion, with the option to withdraw at any time. The study was approved by the Norwegian Regional Committee for Medical and Health Research Ethics (Ref.no: 2014/2057 and 2019/363). Throughout the study, a patient research partner provided input on the burden and relevance of data collected, along with the interpretation and dissemination of the results.

### 2.2 Data collection

#### 2.2.1 Demographics and descriptive clinical characteristics

At baseline, participants self-reported their year of symptom debut. Education level was also self-reported using a scale with seven response options, which we dichotomized according to whether participants had ≥4 years of university/higher education or not. Age (date of birth) and sex were obtained from medical journals. Fulfilment of the ACR criteria for hand OA was assessed by a rheumatologist [17].

At both timepoints, weight and height were measured in kilograms and centimetres, respectively, and utilized to calculate body mass index (kg/m^2^). Participants self-reported on the existence and status of selected comorbidities using a comorbidity index (range: 0–45) [18], (pain) self-efficacy using the Arthritis Self-Efficacy Scale (ASES) (range: 10–100, higher scores indicate better self-efficacy) [19], and whether they were currently working or not. On a figure of both hands, participants marked the hand joints (distal and proximal interphalangeal, metacarpophalangeal, and thumb base joints, n = 30), in which they had experienced pain during the last 6 weeks. Finally, posteroanterior radiographs of both hands were obtained at both timepoints and scored by one experienced reader (IKH) for OA severity. The assessments included the distal and proximal interphalangeal, metacarpophalangeal, first carpometacarpal and scaphotrapeziotrapezoidal joints (n = 32) using a modified Kellgren-Lawrence (KL) scale scored on a 0–4 scale [20]. A radiographic OA sum score was then calculated for each participant (range: 0–128). The percentage of structure-pain concordance for each participant was calculated by dividing the number of hand joints with both radiographic findings (KL ≥ 1) and pain in the last six weeks by the total number of painful hand-joints, multiplied with hundred [21]. As an example, a concordance score of 70 indicated that radiographic findings and pain coexisted in 70 % of the painful joints.

#### 2.2.2 Self-reported IMMPACT model indicator variables

At each timepoint, the severity of fatigue and pain in the hands and the overall body were reported using separate numeric rating scales (NRS, range: 0–10). Neuropathic-like pain quality was evaluated using a modified PainDetect questionnaire [22] (range: 0-38). Anxiodepressive symptoms and pain catastrophizing were assessed by the Hospital Anxiety and Depression Scale sum-score (HADS, range: 0–42) [23], and the Pain Catastrophizing Scale (PCS, range: 0–52) [24], respectively. The sleep question of the 15D-questionnaire (range: 1–5) was used to evaluate sleep [25]. For all self-reported indicator variables, a higher score indicates worse outcomes.

#### 2.2.3 IMMPACT model-indicators from quantitative sensory testing

QST was used as a proxy to assess pain sensitization at baseline and at follow-up [15,16].

Pressure pain thresholds (PPT) (kg/cm^2^) were measured using a handheld digital algometer (FPIX25 Wagner; Wagner Instruments, Greenwich, USA, 1 cm^2^ flat rubber probe), for which PPT was defined as the pressure at which the stimulus was reported by the participant to first change from pressure to slight pain. We used the mean - value from three consecutive pressures at each site separated by 30 s of rest between. Lower pressures indicated higher pain sensitivity. PPT assessments at the midportion of the tibialis anterior muscle served as an indicator of central sensitization. At baseline, PPT assessments at the dorsal side of the most painful interphalangeal joint were used as proxies of peripheral and/or central sensitization [26]. The interphalangeal joint with the most severe swelling and/or bony enlargements was selected if no joints were painful. The same interphalangeal finger joint was assessed at follow-up using the same digital algometer.

At both time points, mechanical temporal summation (TS) was assessed by touching a weighted punctuate probe with a fixed intensity (1 Hz) at the left wrist according to the published protocols [15,16]. Each participant rated his/her NRS pain at the first, fifth, and tenth touch. At baseline, we calculated the TS-delta by subtracting the pain rating of the first touch from the peak of the two other ratings. At follow-up, the test was conducted twice with a 3-min rest in between, and the mean of the two TS-delta values was used in the analyses.

The examinations were conducted by two trained medical students at baseline and by a physician and another student at follow-up. The inter-examiner reliability of the QST was tested at both baseline and follow-up. Nine randomly selected participants at baseline and eleven participants at follow-up were tested by both examiners on the same afternoon. At baseline, the inter-examiner reliability for the two students was moderate with an intraclass correlation coefficient (ICC) (two-way mixed-effects model with absolute agreement, individual measures) of 0.43, 0.44, and 0.56 for the PPT OA-joint, PPT tibialis anterior, and TS-delta, respectively [27]. At follow-up, inter-examiner reliability was good with intraclass correlation coefficients of 0.83, 0.85 and 0.84, respectively [27].

Although measured, CPM was excluded from the current study due to unreliable baseline data (ICC <0.3) and CPM's limited influence in previous analyses [11,15,16].

### 2.3 Statistical analyses

The characteristics of the study population are presented by using frequency proportions, means with standard deviation (SD) or medians with interquartile range (IQR) as appropriate.

Given the potential sex differences in pain sensitization [28], we created sex-standardized PPT and TS values and used these values in the analyses. We subtracted the sex-specific mean from each participant's observed value before dividing by the sex-specific standard deviation (SD), yielding a value representing the number of SDs from the sex-specific average [3,4].

We used histograms and quantile-quantile plots to check for normal distribution across IMMPACT variables, patient characteristics and changes between timepoints. Pearson's correlations were assessed for multicollinearities. Based on preliminary latent class analyses at each timepoint, we identified pain phenotypes and their stability in the main analyses by simultaneously modelling IMMPACT indicators from baseline and follow-up using latent transition analyses (LTA). LTA is a probabilistic, data-driven and person-centred technique used to discover unobserved, or so-called latent subgroups, dependent on the number of classes tested, which allows for tracking fluctuations in (conditional) class membership for each individual, despite missing data [29,30]. We evaluated the optimal number of latent classes ranging from 2 to 6 across the two time points using the R package LMest, which employs the expectation-maximization algorithm to estimate parameters while effectively handling missing values in the data [31,32]. As only 8 participants (3.8 %) had missing data on ≥3 of 20 indicator-values, all participants were included with missingness considered missing completely at random [30]. Due to these aspects, combined with the low sample size, no imputations were done [30]. The Bayesian Information Criterion (BIC) and Aikake Information Criterion (AIC) were used to select the optimal number of classes (lower BIC/AIC is better) [29,30,33]. In addition, criteria of the initial probabilities or class prevalences (≥10 %), and clinical relevance or interpretability were used [11,29,30,33,34]. Model entropy, i.e. the precision in separating classes, with a higher number considered most optimal (range: 0–1), was calculated. Entropy was not used as a selection criterion as overfit models may provide excessively high entropies [30]. As the influence of pain sensitization on OA pain phenotyping is debated, we conducted one model including self-reported IMMPACT variables only (clinical model) in addition to the full model (QST model, comprising all self-reported and QST IMMPACT variables) [9,11]. Participants were allocated to the class with the highest posterior probability at both timepoints, and class differences in indicators were explored both by analysis of variance and visually in a figure using standardized class-specific means. In the figure, PPT values were reversed to ease interpretability. Likewise, between-class differences in demographic and clinical characteristics were explored by ANOVA, Kruskal-Wallis tests or chi^2^-tests [29]. The respective class membership for each participant in the clinical and QST models were compared using weighted Kappa. We conducted two tailed t-tests to compare the changes in indicator variables between participants who transitioned to a more or a less severe pain class relative to their baseline pain levels (worsened or improved, respectively) and those who did not. To ease readability, the raw values of QST (not sex-standardized) were presented in the main tables, while the sex-standardized values used in the analyses were kept in the figures. The LTAs were performed with R version 4.4.2 (https://www.R-project.org/) (Supplementary Table 7), while Stata/CE v.18 basic edition was used for the remaining analyses and demographic descriptions.

## 3 Results

### 3.1 Sample characteristics

Of the 300 participants at baseline, 213 (71 %) completed the follow-up examinations after an average of 3.5 years (range: 2.4–4.2 years) (Supplementary Fig. 1). Most baseline characteristics were similar among those with versus those without follow-up data, except for fatigue being higher in participants who were lost to follow-up (Supplementary Table 1). At baseline, the mean age (standard deviation, SD) of the included participants was 60.9 (6.0) years, and the majority (87 %) were women (Table 1). The mean NRS hand-pain was 3.7 (2.1), with n = 66 (31.1 %) participants reporting pain intensity above the patient-acceptable symptom score of 4 NRS points [35].

**Table 1:** Baseline participant characteristics (QST model).

Characteristics	Sample (n = 213)	Class 1 n = 81 (38.0 %)	Class 2 n = 49 (23.0 %)	Class 3 n = 58 (27.2 %)	Class 4 n = 25 (11.7 %)	P value
***Demographics and clinical characteristics***						
Age, mean (SD) years	60.9 (6.0)	61.0 (6.0)	61.9 (5.5)	60.9 (6.4)	58.2 (5.7)	0.09
Sex, n (%) women	185 (86.9)	66 (81.5)	44 (90.0)	53 (91.4)	22 (91.7)	0.32
Higher education, n (%)	130 (61.0)	62 (76.5)	33 (67.3)	24 (41.4)	11 (44.0)	0.001
Working, n (%)^a^	124 (58.7)	59 (72.8)	27 (55.1)	27 (46.6)	11 (44.0)	0.002
Fulfilment of ACR hand OA criteria, n (%)	202 (94.8)	76 (93.8)	45 (91.8)	57 (98.3)	24 (96.0)	0.47
BMI, mean (SD) kg/m^2^	26.6 (4.8)	25.5 (3.9)	26.6 (5.6)	28.0 (4.6)	26.7 (4.8)	0.02
Symptom duration, median (IQR) years^a^	6 (3–13)	4 (2–10)	6.5 (2–11.5)	8 (4–17)	6 (4–13)	0.02
Comorbidity index sum score (range: 0–45), mean (SD)	7.5 (4.1)	5.7 (3,6)	7.8 (3.8)	8.6 (4.0)	10.2 (4.1)	0.001
Arthritis self efficacy scale (range: 10–100), mean (SD)^a^	63.2 (15.8)	71.8 (13.5)	65.5 (13.3)	55.0 (13.2)	49.5 (15.0)	0.001
KL sum score (range: 0–128), mean (SD)	30.8 (18.5)	29.3 (17.4)	30.8 (18.2)	36.2 (18.9)	22.7 (19.0)	0.009
Structure-pain concordance (%), mean (SD)	60.9 (36.8)	56.6 (40.9)	63.1 (38.1)	71.5 (27.4)	46.2 (33.1)	0.02
***IMMPACT indicators***						
NRS hand pain (range: 0–10), mean (SD)^a^	3.7 (2.1)	2.2 (1.4)	3.1 (1.4)	5.3 (1.9)	6.0 (1.9)	0.001
NRS all bodily pain (range: 0–10), mean (SD)^a^	4.0 (2.3)	2.2 (1.3)	4.4 (2.1)	5.1 (2.0)	6.3 (1.7)	0.001
Sleep (15D) (range:1–4), mean SD	2.25 (1.0)	1.8 (0.8)	1.9 (0.7)	2.9 (0.9)	3.0 (0.9)	0.001
NRS fatigue, (range: 0–10), mean (SD)^a^	3.8 (2.8)	2.2 (2.4)	3.5 (2.3)	5.1 (2.2)	6.9 (2.2)	0.001
Hospital anxiety depression scale (range: 0–42), mean (SD)^a^	6.9 (5.5)	3.1 (2.2)	9.6 (3.8)	5.3 (2.9)	17.5 (4.2)	0.001
Pain catastrophizing scale (range: 0–52), mean (SD)^a^	10.8 (7.7)	5.8 (4.8)	13.2 (7.5)	11.9 (6.3)	19.5 (7.5)	0.001
Neuropathic like pain (PainDetect) (range: 1-38), mean (SD)	9.5 (6.0)	5.8 (4.0)	7.7 (3.9)	13.5 (5.6)	15.9 (5.4)	0.001
PPT OA joint, mean (SD) kg/cm^2^†	3.9 (1.9)	4.2 (1.9)	4.0 (2.1)	3.6 (1.8)	3.4 (1.9)	0.20
PPT m. tibialis anterior, mean (SD) kg/cm^2^^b^	5.6 (2.7)	5.7 (2.6)	6.0 (3.0)	5.4 (2.5)	4.6 (2.4)	0.14
Mechanical temporal summation, mean (SD) NRS change^b^	1.5 (1.6)	1.3 (1.4)	1.3 (1.4)	1.7 (1.6)	2.6 (1.9)	0.001

*Abbreviations*: ACR = American College of Rheumatology; OA = osteoarthritis; SD = standard deviation; IQR = interquartile range; BMI = body mass index; ASES = arthritis self-efficacy scale; AUSCAN = Australian/Canadian Osteoarthritis Hand Index; KL = Kellgren-Lawrence; IMMPACT = Initiative on Methods, Measurement, and Pain Assessment in Clinical Trials; IMMPACT= Initiative on Methods, Measurement, and Pain Assessment in Clinical Trials; NRS = Numerical Rating Scale; HADS = Hospital Anxiety and Depression Scale; PCS = Pain Catastrophizing Scale; PPT = pain pressure threshold.

Missing values.

a participant characteristics: Work (n = 2); symptom duration (n = 15); ASES (n = 5); NRS hand pain (n = 1); NRS all bodily pain (n = 3); NRS fatigue (n = 3); HADS (n = 7); PCS (n = 3); PPT OA joint (n = 5), PPT m. tibialis anterior (n = 5); temporal summation (n = 1).

b to ease interpretability, the table presents raw QST values. In the analyses, results were derived using the sex-standardized QST values.

### 3.2 Model selection

A four-class model was selected. Each fit statistic (BIC and AIC) preferred five classes in both the QST model and the clinical model, followed by four classes (Supplementary Tables 2 and 3). However, the initial probabilities indicated that at least one out of five classes would include <10 % of the sample. Furthermore, the conditional response means (clinical relevance) were deemed less discrete across classes. In contrast, the four-class model satisfied all the preset model selection criteria and was deemed optimal, with fit statistics being lower (i.e., better fit) for the clinical model compared to the QST model. Both models demonstrated similar degrees of high precision, with entropies of 0.84 and 0.86 for the clinical and the QST model, respectively. The average posterior probabilities were >0.91 for both models at each timepoint, with confidence intervals showing that the participants had an overall high probability of belonging to their allocated class (Supplementary Tables 2 and 3). The agreement between the two models was high with a weighted kappa value of 0.91 for class memberships.

### 3.3 Class characteristics

**Fig. 1:**
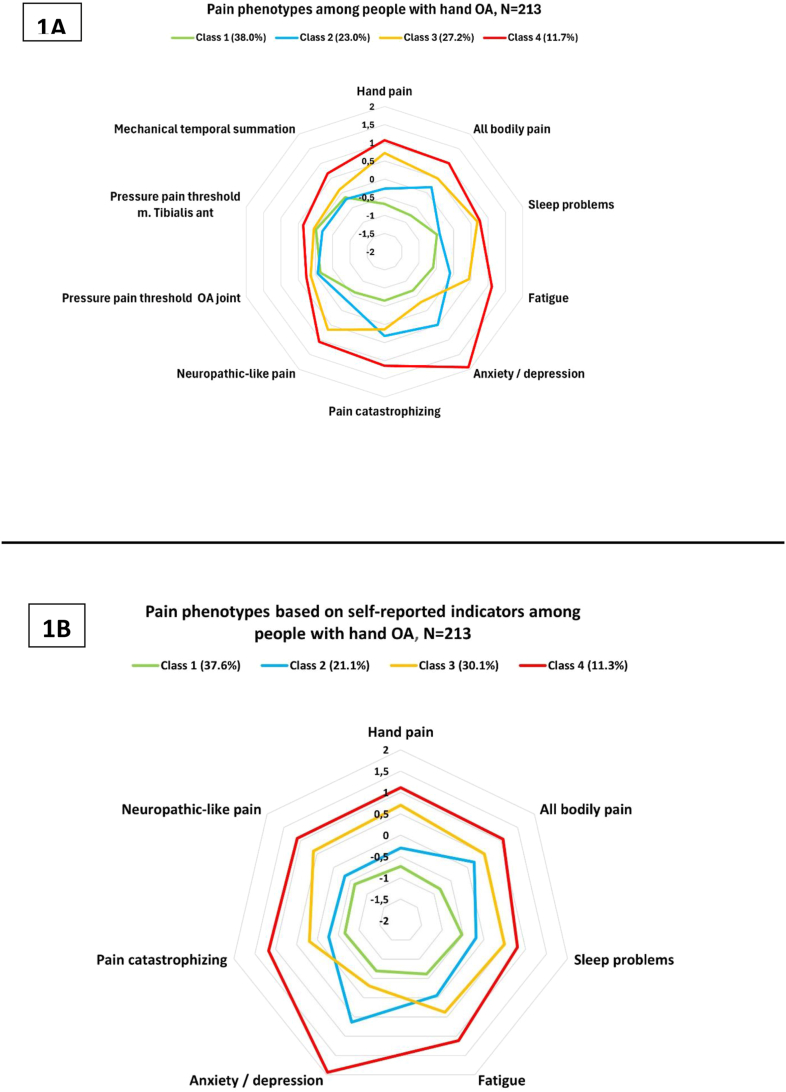
Characteristics of pain phenotypes using all available IMMPACT variables (QST model) (1A), and self-reported indicators only (clinical model) (1B), presented using standardized values. Percentages in brackets indicate class prevalences (the size of each class). To ease interpretability, measures of pressure pain thresholds have been reversed. In both spider plots 1A and 1B (models), Class 1 (i.e., around 38.0 % of the sample) is characterized by the lowest severities across all indicators relative to the other classes, while Class 4, the smallest class, exhibits the relatively highest severities (unfavorable outcomes). Compared to Class 1, Class 2 exhibits more severe anxiodepressive symptoms and greater bodily pain. Finally, Class 3 closely resembles Class 4, but with a less severe psychological burden, including milder anxiodepressive symptoms and pain catastrophizing.

For the QST model, all indicators except for PPT showed statistically significantly differences across the classes (p < 0.05) (Table 1, Fig. 1A). At baseline, Class 1 (n = 81, 38.0 %) was recognized by low scores on most indicators relative to the other classes (i.e., least severely affected). In contrast, Class 4 (n = 25, 11.7 %) demonstrated an overall high severity on all indicators. Compared to Class 1, Class 2 (n = 49, 23.0 %) differed with respect to more severe anxiodepressive symptoms, pain catastrophizing, and overall bodily pain. Finally, Class 3 was similar to Class 4 except for having less severe anxiodepressive symptoms, pain catastrophizing and temporal summation (Fig. 1A). The relative structure and meaning of classes remained constant at follow-up (data not shown).

The classes in both models were generally well separated by demographic and clinical characteristics (Table 1). For example, self-efficacy and education levels decreased when comparing classes 1 through 4. Although classes 3 and 4 exhibited high levels of hand pain relative to the other classes, Class 4 demonstrated the lowest overall radiographic severity and the lowest proportion of radiographic findings in the painful joints (Table 1).

The clinical model produced results comparable to those presented for the QST model (Table 1, Fig. 1B).

### 3.4 Class stability

**Fig. 2:**
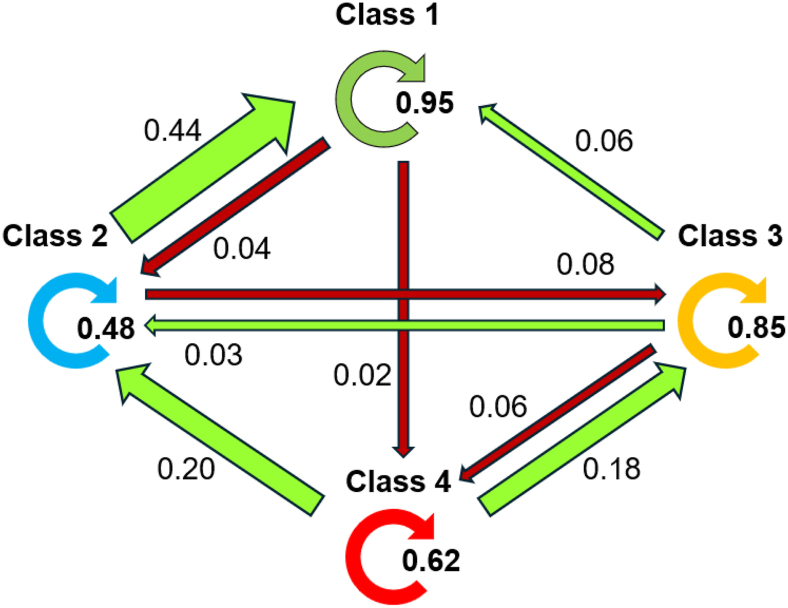
Transition probabilities of pain phenotypes in hand OA (QST model), N = 213. Light green colored arrows indicate the probabilities of transitioning to a class with less severe pain (improvement), while dark red arrows indicate the probabilities of transitioning to a class with more severe pain (worsening), conditional on the class membership at baseline. Circular arrows indicate the probability of remaining in the respective baseline class after 3.5 years. For example, the probability of remaining in Class 2 after 3.5 years was 0.48, while the probability of transitioning from Class 2 to Class 1 (improvement) or Class 3 (worsening) was 0.44 and 0.08, respectively.

Overall, for both models, Class 1 was the most stable with an estimated 95% probability of remaining in this class at follow-up (Fig. 2). In contrast, classes 2 and 4 were the least stable, with relatively higher total probabilities of transferring to another class.

At follow-up, 169 participants (79.3 %) in the QST model and 168 (78.9 %) in the clinical model remained in their baseline class (Table 2 and Supplementary Table 4). Among those transitioning to another class, the majority improved (QST model: 72.7 %, and clinical model: 80.0 %) (Table 2 and Supplementary Table 4). Compared to those not transitioning, participants transitioning to a class reporting less severe pain generally experienced greater improvements in anxiodepressive symptoms, pain catastrophizing, or fatigue in addition to pain. Conversely, those transitioning to a class reporting more severe pain showed greater worsening in anxiodepressive symptoms or sleep and fatigue (Table 2 and Supplementary Tables 4–6).

**Table 2:** Absolute changes in indicators and clinical characteristics among participants transitioning between classes versus those remaining in their baseline class throughout follow-up (QST model).

Measures	Sample (n = 213)	Same class (n = 169)	Better (n = 32)	Worse (n = 12)
***Changes in clinical characteristics***				
BMI, mean (SD) kg/m^2^	0.7 (2.1)	0.6 (2.3)	1.0 (1.7)	0.7 (2.5)
ASES (range: 10–100), mean (SD)	0.6 (18.1)	1.2 (18.4)	1.0 (17.3)	−7.7 (16.2)
Comorbidity index sum score (range: 0–45), mean (SD)	−0.7 (3.5)	−0.5 (3.4)	−1.8 (3.3)^a^	−0.8 (4.5)
KL sum score (range: 0–128), mean (SD)	2.6 (2.7)	2.7 (2.8)	2.3 (2.4)	2.4 (1.9)
Structure-pain concordance, mean (SD) %	−4.3 (42.8)	−4.4 (39.4)	−3.4 (54.1)	−4.6 (57.0)
**Changes *IMMPACT indicators***				
NRS hand pain (range: 0–10), mean (SD)	−0.4 (2.2)	−0.4 (2.1)	−1.3 (2.0)^a^	1.4 (2.7)^a^
NRS all bodily pain (range: 0–10), mean (SD)	−0.4 (2.4)	−0.0 (2.0)	−2.8 (2.7)^a^	2.0 (2.5)^a^
Sleep (15D) (range:1–4), mean SD	−0.1 (0.9)	−0.1 (0.9)	−0.2 (1.1)	0.2 (0.8)
NRS fatigue, (range: 0–10), mean (SD)	−0.9 (2.6)	−0.8 (2.5)	−1.9 (2.9)^a^	1.0 (1.5)^a^
Depression/Anxiety (HADS) (range: 0–42), mean (SD)	−0.9 (4.2)	−0.4 (3.1)	−4.8 (4.9)^a^	2.3 (7.5)^a^
Pain catastrophizing (PCS) (range: 0–52), mean (SD)	−2.7 (7.3)	−1.8 (6.4)	−8.5 (8.1)^a^	0.9 (9.0)
Neuropathic like pain (PainDetect) (range: 1-38), mean (SD)	0.1 (5.1)	0.3 (5.0)	−1.9 (5.5)^a^	2.8 (4.8)^a^
PPT OA joint, mean (SD) kg/cm^2^^b^	−0.3 (1.8)	−0.3 (1.8)	0.2 (2.1)	−0.9 (1.3)
PPT m.tibibalis anterior, mean (SD) kg/cm^2^^b^	−0.7 (2.4)	−0.7 (2.5)	−0.4 (2.5)	−0.5 (2.2)
Mechanical temporal summation, mean (SD) NRS^b^	0.4 (2.0)	0.44 (1.9)	0.3 (2.7)	0.8 (1.8)

*Abbreviations:* BMI = body mass index; SD = standard deviation; ASES = arthritis self-efficacy scale; AUSCAN = Australian/Canadian Osteoarthritis Hand Index; KL = Kellgren-Lawrence; IMMPACT= Initiative on Methods, Measurement, and Pain Assessment in Clinical Trials; NRS = numeric rating scale; PPT = pressure pain threshold.

a Statistically significantly different at p < 0.05 versus participants not transitioning.

b to ease interpretability, the table presents raw QST values. In the analyses, results were derived using the sex-standardized QST values.

## 4 Discussion

Our study is the first to explore pain phenotypes and their stabilities among persons with hand OA. Defining classes by self-reported pain, psychological factors, sleep, and fatigue aligned well with models that also incorporated QST. At follow-up, most participants retained their baseline class, with the largest stability observed in the class with the least severe pain. Among those who transitioned to another class, about three-quarters transitioned to a class characterized by less pain and psychological burden.

The classes identified may be clinically distinct, supporting an impact of including various self-reported variables. For example, in the QST model, Classes 3 and 4 both had substantially more severe hand OA pain compared to Classes 1 and 2 (mean values: 5.3–6.0 vs 2.2–3.1). Notable, this difference exceeds the minimally clinically important difference of 1–2 points on a 0–10 scale (Table 1) [36]. Furthermore, classes 3 and 4 exhibited hand pain above the NRS patient-acceptable symptom score threshold of 4 points and were recognized by more severe sleep problems and high fatigue than classes 1 and 2 [35]. However, between-group differences were also observed among other classes. For example, compared to classes 1 and 3, more severe anxiodepressive symptoms were observed in classes 2 and 4. In our study, the average class-specific levels of pain catastrophizing ranged from 5.8 to 19.5 points, indicating that none of the classes exceeded the previously suggested cut-off of 30 points for clinically relevant levels of pain catastrophizing [5]. However, previous analyses of this study population have shown that also lower levels of pain catastrophizing relate to pain, suggesting that levels below the proposed cut-off may be clinically relevant [4].

The results of the QST model and clinical model suggest that QST measures of pain sensitization are less influential in class separation than clinical variables in persons with established disease. Although pain phenotyping within hand OA research is limited, our results may be compared to those of knee OA [9,37,38]. In line with our results, a cross-sectional knee OA study using the IMMPACT framework in first-time orthopaedic consultations identified classes that differed with regards to pain severity, self-reported measurements of i.e., psychological status, sleep, fatigue, and TS, while the differences in PPT and CPM were small [11]. Similarly, two studies on community-dwelling older adults with knee OA supported our findings, including a minimal impact from PPT to identify classes [37,38]. Conversely, studies including people at risk of knee OA or being in early stages of disease found PPT to be more important [9,34,39]. In overall, QST measures of pain sensitization may be more useful in identifying pain phenotypes in early stages of the disease. For people with established disease, the QST influence on pain has probably already occurred. However, comparisons with our participants may be limited due to the unique nature of hand OA, which is a non-weightbearing condition affecting multiple joints, often at varying structural and symptomatic stages [21,40]. Furthermore, we acknowledge that the baseline reliability of QST in our study limit conclusions.

Nevertheless, the various influence of psychosocial factors may indicate different pain mechanisms between the classes, requiring tailored interventions. While the PainDetect sum-score in Class 4 suggests possible neuropathic-like pain (Table 1), recent evidence suggests that the overall high pain and limited structural hand joint pathology (both overall and in painful joints) alongside a high psychosocial comorbidity burden may instead reflect nociplastic pain driven by central mechanisms [6,22,41,42]. Although QST measures influenced class separation to a minimal extent, the higher degree of TS in Class 4 may strengthen this argument. Class 4 may require a multidimensional treatment approach, including attention to sleep and psychological symptoms [6,7,37,41,43]. In contrast, classes 1 and 2, characterized by less severe pain, radiographic findings in most symptomatic joints, and higher self-efficacy, may benefit from treatments targeting peripheral and nociceptive factors [11,44,45]. Class 3 was highly comparable to Class 4, except for less anxiodepressive symptoms and TS. Additionally, Class 3 exhibited the most severe structural damage on radiographs and high concordance rate of radiographic findings in painful joints. Studies support a relationship between peripheral factors (i.e., radiographic findings) and pain in the same joint, especially in more advanced stages, pointing to a higher nociceptive component for this class [12,21,46]. Given the complex or bidirectional associations between pain and factors like high BMI, sleep problems, and low self-efficacy, a possible systemic contribution to this ongoing pain may also co-exist in Class 3 (Table 1), yet research addressing these factors in hand OA pain is lacking [1,14,45,47].

We found that the probability of remaining in the baseline class ranged from 0.48 to 0.95, with the lowest probabilities observed in the smallest classes (Fig. 2). Although pain in hand OA may fluctuate, indeed, most participants remained in their baseline class at follow-up, showing minimal changes in their overall indicator severities. This finding aligns well with previously reported results from studies on knee OA or different hand complaints including hand OA, where 70–86 % remained in the same class, even after longer period of follow-up [9,10]. Given our small sample size, the impact of longitudinal changes in class indicators on transitions between specific classes was not assessed [29,33]. However, on group-level, participants transitioning between classes collectively showed generally larger changes in pain and psychosocial factors than participants who did not transition (Table 2). Our findings contrast the results from a study of people at risk of knee OA, where pain sensitization rather than psychosocial factors influenced between-class transitions [9]. The divergent results may be explained by different study populations with regards to established vs. early disease, as previously discussed. Our finding of a large proportion of participants transitioning to less severe pain class compared to their baseline—including those initially in the most severe class—aligns with previous findings in individuals with various hand complaints, including hand OA [10]. Possible explanations of this finding may be treatments received between the visits, natural disease fluctuations and better disease coping. In addition, regression to the mean cannot be excluded, as participants were likely referred to secondary care during periods of heightened symptom severity.

The changes in radiographic findings among participants transitioning between classes were not significantly different from those of non-transitioning participants (Table 2), raising questions about the role of joint structures in defining class memberships. Previous studies assessing relationships between overall radiographic changes and changes in pain in hand OA support that these associations are weak [9,48]. Although relationships between longitudinal changes in pain severity and radiographic severity at the joint level have been shown, these analyses were adjusted for age and sex only, limiting direct comparison [49]. It remains unclear whether changes in synovitis or bone marrow lesions, both possibly linked to pain, may have influenced transitions in our study [50].

Our study has some limitations. Firstly, our results may have a limited generalizability to men and people attending primary care. Secondly, the results should be interpreted cautiously, as the phenotypes described are probabilistically derived constructs vulnerable to sample size, indicators modelled and the recall bias observed in self-reported measurements [29,30]. Despite using IMMPACT, influences of unmeasured indicators or covariates are highly possible. While the ideal sample size for LTA remains undefined, we acknowledge that our sample size is relatively small, posing a potential bias risk in class estimation despite the high posterior probabilities and entropy observed [33]. Changes in QST reliability between visits may affect confidence in our results. However, ICC estimates could be unreliable due to the low number of tests. Also, previous studies from this study population found minimal influence on results by using QST data from the assessor performing most tests at baseline in sensitivity analyses, compared to data from both assessors [4,16,47]. We recommend that the identification and stability of pain phenotypes in hand OA should be further explored in other samples for external validation, preferably by using larger samples and multiple time-points. Finally, future studies should explore whether different pain phenotypes vary in their response to symptomatic treatments for hand OA.

In conclusion, distinct pain phenotypes with various stabilities, potentially driven by different pain mechanisms and with varying implications for treatment likely exist among persons with hand OA in secondary care. Psychosocial factors, rather than pain sensitization, appear to play a key role in defining these phenotypes, and influencing transitions between them.

## Author contributions

Substantial contributions to the conception and design of the study: DHB, MG, EM, TN, IK and IKH, or acquisition/analysis of data: DHB, LMS, PSP, MG, EM, and IKH. Interpretation of data and drafting of the manuscript or revising it critically: DHB, LMS, PSP, MG, EM, IK, TN and IKH. Final approval of the publication: DHB, LMS, PSP, MG, EM, TN, IK and IKH. Agreement to be accountable for all aspects of the work in ensuring that questions related to the accuracy or integrity of any part of the work are appropriately investigated and resolved: DHB, EM, PSP, MG, LMS, TN, IK and IKH.

## Funding

The Nor-Hand study was funded by the Norwegian Research Council (project number: 328657), ADVANCE grant from Pfizer/Lily, South-East Norway Regional Health Authority, Pahle's foundation, Simon Fougner Hartmann's Family foundation and Trygve Gythfeldt's research foundation. TN was supported by NIH K24 AR070892, P30 AR072571 and R01 AG066010. DHB's work was completely funded by his grant from the Norwegian Women's Public Health Association's department at Haugesund Rheumatological Hospital, Haugesund, Norway, to support his PhD-programme. The funders were not involved in the study design, collection, analysis or interpretation of the current data.

## Declaration of competing interest

For transparency, IKH receives consulting fees (honoraria for lectures) from Argenx, Pacira, GSK, Grünenthal and Abbvie. She also reports positions in Osteoarthritis Research Society Internationale (OARSI); Chair of guidelines committee (unpaid) and Chair of EsSKOA steering committee (unpaid). TN reports on consulting fees from Novartis outside the current work. DHB, LMS, MG, EM, PSP and IK have nothing to disclose.
