# Supplementary material for: The stability of pain phenotypes in people with hand osteoarthritis – results from the NOR-HAND study
Source: Osteoarthr Cartil Open. 2026 Jan 14;8(1):100745. doi: 10.1016/j.ocarto.2026.100745 (PMC12861019; doi:10.1016/j.ocarto.2026.100745)
Supplement: Multimedia component 1 [file mmc1.docx]

**Supplementary Figure 1** (for this purpose: Screenshot only): Flowchart illustrating the inclusion of participants in the current study


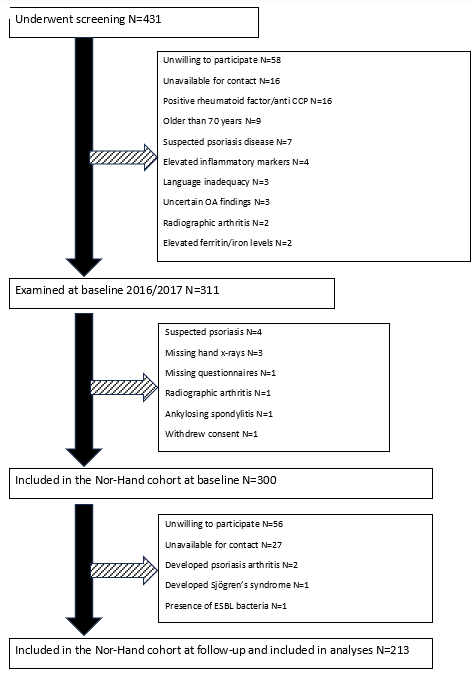


**Supplementary Table 1:** Differences in baseline characteristics between participants attending the baseline assessments (2016/2017) only versus participants attending both visits (2016/2017 and 2019-20121).

| Characteristic | Values | | |
| --- | --- | --- | --- |
|  | **Baseline only (n=87)** | **Both visits (n=213)** | **P-value** |
| *Clinical characteristics* |  |  |  |
| Age, mean (SD) | 60.5 (6.6) | 60.9 (6.0) | 0.69 |
| Sex, n (%) women | 81 (93.1) | 185 (86.9) | 0.12 |
| Higher education and/or University, n (%) | 45 (51.7) | 129 (60.5) | 0.16 |
| Working, n (%) | 36 (41.4) | 124 (58.2) † | 0.01‡ |
| Body Mass Index, mean (SD) kg/m^2^ | 26.4 (5.4) | 26.6 (4.8) | 0.65 |
| Symptom duration y, median (IQR) | 6 (3 – 13)* | 6 (3 – 13) † | 0.79 |
| Fulfils ACR hand OA criteria, n (%) | 75 (87.2) | 202 (94.8) | 0.02‡ |
| ASES (range 10-100), mean (SD) | 63.6 (23.2)* | 64.4 (22.9) † | 0.79 |
| Comorbidity index sum score (range: 0-45), mean (SD) | 8.3 (4.6) | 7.5 (4.1) | 0.11 |
| KL sum score (range: 0-128), mean (SD) | 27.8 (19.8) | 30.8 (18.6) | 0.22 |
| *IMMPACT indicators* |  |  |  |
| NRS hand pain (range: 0–10), mean (SD) | 4.0 (2.4) | 3.7 (2.2) † | 0.29 |
| NRS all bodily pain (range: 0-10), mean (SD) | 4.2 (2.3) | 4.0 (2.3) † | 0.37 |
| NRS fatigue (range: 0-10), mean (SD) | 4.6 (3.2)* | 3.8 (2.8) † | 0.04‡ |
| Sleep problems (15D) (range: 1-5), mean (SD) | 2.4 (1.1)* | 2.3 (1.0) | 0.23 |
| Neuropathic like pain (PainDetect) (range: 0-38), mean (SD) | 10.1 (6.7) | 9.1 (5.8) | 0.23 |
| HADS total sum score (range: 0–42), median (IQR) | 7 (2-12)* | 6 (3-10) † | 0.32 |
| PCS total sum score (range: 0–52), median (IQR) | 9.5 (5-15)* | 9.0 (5-15) † | 0.93 |
| PPT OA joint, mean (SD) Kg/cm^2^ | 3.9 (1.9)* | 3.9 (1.9) † | 0.99 |
| PPT m. tibialis anterior, mean (SD) Kg/cm^2^ | 5.5 (2.3)* | 5.6 (2.7) † | 0.83 |
| Mechanical Temporal summation, median (IQR) | 1 (0 -3)* | 1 (0 -2) † | 0.40 |
|  |  |  |  |

*Abbreviations*: ACR = American College of Rheumatology OA = osteoarthritis; SD = standard deviation; IQR = interquartile range; ASES = arthritis self-efficacy scale; AUSCAN = Australian/Canadian Osteoarthritis Hand Index; KL = Kellgren-Lawrence; IMMPACT = Initiative on Methods, Measurement, and Pain Assessment in Clinical Trials; NRS = Numerical Rating Scale; HADS = Hospital Anxiety and Depression Scale; PCS = Pain Catastrophizing Scale; PPT= pain pressure threshold.

*Missing values*:

* = Baseline only: Symptom duration (n=5); ASES (n=1); NRS fatigue (n=1); Sleep (n=1), HADS (n=2), PCS (n=1); PPT OA joint (n=4); PPT m.tibialis anterior (n=4), temporal summation (n=1)

† = Both visits: Work (n=2); Symptom duration (n=15); ASES (n=5), NRS hand pain (n=1), NRS all bodily pain (n=3); NRS fatigue (n=3); HADS (n=7), PCS (n=3); PPT OA joint (n=5), PPT m.tibialis anterior (n=5), temporal summation (n=1)

‡=Differences with P<0.05.

**Supplementary Table 2**

Output from latent transition analyses on pain phenotypes using the complete set of indicators from the IMMPACT framework (QST model), N=213

| **K** | **BIC** | **AIC** | **LL** | **Entropy** | **Initial probabilities** | **Average posterior probability (95% CI)** | **Average posterior probability for class at BL**  **(95% CI)** | **Average posterior probability for class at FU**  **(95% CI)** |
| --- | --- | --- | --- | --- | --- | --- | --- | --- |
| 2 | 17643.9 | 17381.7 | -8612.8 | 0.805 | 0.576  0.424 | 0.946 (0.934, 0.956) | 0.947 (0.933, 0.961) | 0.945 (0.930, 0.959) |
| 3 | 17644.9 | 17332.3 | -8573.1 | 0.835 | 0.343  0.511  0.146 | 0.928 (0.917, 0.939) | 0.933 (0.918, 0.948) | 0.923 (0.907, 0.939) |
| 4 | 17628.8 | 17259.0 | -8519.5 | 0.857 | 0.368  0.239  0.275  0.119 | 0.925  (0.913, 0.937) | 0.928  (0.912, 0.945) | 0.922  (0.905, 0.940) |
| 5 | *17623.2* | 17189.6 | -8465.8 | 0.862 | 0.374  0.090  0.187  0.264  0.086 | 0.922 (0.910, 0.935) | 0.931 (0.915, 0.947) | 0.914 (0.895, 0.932) |
| 6 | 17720.3 | 17216.1 | -8458.1 | 0.872 | 0.114  0.317  0.194  0.231  0.097  0.048 | 0.921 (0.906, 0.930) | 0.914 (0.896, 0.932) | 0.921 (0.906, 0.937) |

*Abbreviations*: K= number of classes tested; BIC= Bayesian Information Criterion; AIC= Aikake Information Criterion; LL=log-likelihood; CI= confidence interval

**Supplementary Table 3**

Output from latent transition analyses on pain phenotypes using only self-reported indicators from the IMMPACT framework (Clinical model), N=213

| **K** | **BIC** | **AIC** | **LL** | | **Entropy** | | **Initial**  **Probabilities** | | **Average posterior probability (95% CI)** | **Average posterior probability for class at BL (95% CI)** | **Average posterior probability for class at FU (95% CI)** |
| --- | --- | --- | --- | --- | --- | --- | --- | --- | --- | --- | --- |
| 2 | 14125.8 | 13974.6 | | -6942.3 | | 0.803 | | 0.567  0.433 | 0.944 (0.933, 0.954) | 0.944 (0.929, 0.960) | 0.943 (0.928, 958) |
| 3 | 14091.4 | 13899.4 | | -6892.9 | | 0.823 | | 0.530  0.306  0.164 | 0.923 (0.911, 0.936) | 0.922 (0.905, 0.940) | 0.924 (0.907, 0.942) |
| 4 | 14092.8 | 13854.2 | | -6856.1 | | 0.844 | | 0.373  0.210  0.304  0.113 | 0.917 (0.904, 0.930) | 0.924 (0.907, 0.941) | 0.910 (0.891, 0.930) |
| 5 | *14057.0* | 13764.8 | | -6795.4 | | 0.872 | | 0.374  0.145  0.088  0.288  0.106 | 0.923 (0.910, 0.935) | 0.928 (0.912, 0.945) | 0.917 (0.899, 0.936) |
| 6 | 14177.5 | 13824.5 | | -6807.3 | | 0.827 | | 0.232  0.197  0.193  0.161  0.110  0.107 | 0.880 (0.866, 0.895) | 0.889 (0.869, 0.909) | 0.871 (0.850, 0.823) |

*Abbreviations*: K= number of classes tested; BIC= Bayesian Information Criterion; AIC= Aikake Information Criterion; LL=log-likelihood; CI= confidence interval

**Supplementary Table 4**: Absolute changes in indicators and clinical characteristics among participants transitioning between classes versus those remaining in their baseline class throughout follow-up (Clinical model), N=213.

| **Measure** | **Sample (n=213)** | **Same class**  **(n=168)** | **Better**  **(n=36)** | **Worse**  **(n=9)** |
| --- | --- | --- | --- | --- |
| ***Changes in clinical characteristics*** |  |  |  |  |
| BMI, mean (SD) kg/m^2^ | 0.7 (2.1) | 0.6 (2.1) | 1.1 (2.6) | 0.0 (2.4) |
| ASES (range: 10-100), mean (SD) | 0.6 (18.1) | 1.2 (18.0) | 2.4 (15.7) | -15.8 (22.3)* |
| Comorbidity index sum score (range: 0-45), mean (SD) | -0.7 (3.5) | -0.5 (3.4) | -1.7 (3.7)* | -1.4 (3.8) |
| KL-sum score (range: 0-128), mean (SD) | 2.6 (2.7) | 2.6 (2.6) | 2.4 (2.6) | 4.9 (5.3) |
| Structure-pain concordance , mean (SD) % | -4.3 (42.8) | -4.1 (41.1) | -4.9 (49.3) | -5.2 (51.1) |
| **Change *IMMPACT indicators*** |  |  |  |  |
| NRS hand pain (range: 0-10), mean (SD) | -0.4 (2.2) | -0.4 (2.1) | -1.2 (2.2)* | 1.7 (2.7)* |
| NRS all bodily pain (range: 0-10), mean (SD) | -0.4 (2.4) | -0.1 (2.2) | -2.2 (2.1)* | 2.8 (2.3)* |
| Sleep (15D) (range:1-4), mean SD | -0.1 (0.9) | -0.1 (0.9) | -0.1 (0.9) | 0.4 (0.5)* |
| NRS fatigue, (range: 0-10), mean (SD) | -0.9 (2.6) | -0.8 (2.6) | -1.7 (2.7) | 0.2 (2.1) |
| Depression /Anxiety (HADS) (range: 0-42), mean (SD) | -0.9 (4.2) | -0.3 (3.1) | -4.8 (4.9)* | 3.2 (7.5)* |
| Pain catastrophizing (PCS) (range: 0-52), mean (SD) | -2.7 (7.3) | -2.0 (3.1) | -7.1 (7.4)* | 2.2 (6.9) |
| Neuropathic like pain (PainDetect) (range: 0-38), mean (SD) | 0.1 (5.1) | 0.3 (5.0) | -1.5 (5.9) | 2.6 (3.4) |

*Abbreviations:* BMI = body mass index; SD = standard deviation; ASES = arthritis self-efficacy scale; AUSCAN = Australian/Canadian Osteoarthritis Hand Index; KL = Kellgren-Lawrence; IMMPACT= Initiative on Methods, Measurement, and Pain Assessment in Clinical Trials; NRS = numeric rating scale

* = Statistically significantly different at p<0.05 versus participants not transitioning

**Supplementary Table 5:**

Baseline indicator and clinical characteristics of participants transitioning (improved or worsened) versus those remaining in the same class throughout the follow-up period, results from the QST model (N=213).

| **Measure** | **Sample**  **(n=213)** | **Same Class**  **(n=168, 78.9%)** | **Improved**  **(n=32, 15.0%)** | **Worsened**  **(n=12, 5.6%)** |
| --- | --- | --- | --- | --- |
| ***Clinical characteristics*** |  |  |  |  |
| Age, mean (SD) years | 60.9 (6.0) | 60.7 (6.2) | 61.5 (5.2) | 61.2 (6.1) |
| No of women, n (%) | 185 (86.9) | 151 (89.9) | 25 (78.2) | 9 (75.0) |
| Higher education, n (%) | 130 (61.0) | 106 (62.7) | 17 (53.1) | 7 (58.3) |
| Working, n (%) | 124 (58.8) | 97 (57.7) | 17 (53.2) | 10 (83.3) |
| Fulfils ACR hand criteria, n (%) | 202 (94.8) | 161 (95.3) | 29 (90.6) | 12 (100) |
| BMI, mean (SD) kg/m^2^ | 26.6 (4.8) | 26.5 (4.8) | 27.7 (5.1) | 25.8 (3.3) |
| Symptom duration (y), median (IQR) years | 6 (3-13) | 6 (2-13) | 7 (3-13) | 7 (5-14.5) |
| Comorbidity index sum score (range: 0-45), mean (SD) | 7.5 (4.1) | 7.4 (4.1) | 8.5 (4.2) | 6.4 (3.5) |
| ASES (range: 10-100), mean (SD) | 63.2 (15.8) | 64.1 (15.7) | 58.4 (16.2) | 64.8 (14.7) |
| KL-sum score (range: 0-128), mean (SD) | 31.3 (18.9) | 31.8 (19.3) | 28.6 (18.0) | 32.1 (15.0) |
| Structure-pain concordance, mean (SD) % | 60.9 (36.8) | 60.7 (37.3) | 59.1 (34.5) | 68.7 (37.6) |
| ***IMMPACT Indicators*** |  |  |  |  |
| NRS hand pain (range: 0-10), mean (SD) | 3.7 (2.1) | 3.6 (2.3) | 4.2 (2.1) | 2.9 (1.2) |
| NRS all bodily pain (range: 0-10), mean (SD) | 4.0 (2.3) | 3.7 (2.1) | 5.8 (2.5)* | 2.8 (1.2) |
| Sleep problems (15D) (range:1-4), mean SD | 2.25 (1.0) | 2.2 (1.0) | 2.3 (1.0) | 2.4 (0.9) |
| NRS fatigue, (range: 0-10), mean (SD) | 3.8 (2.8) | 3.6 (2.8) | 4.8 (3.0)* | 3.7 (2.4) |
| Depression /Anxiety (HADS) (range: 0-42), mean (SD) | 6.9 (5.5) | 5.9 (5.3) | 10.9 (5.2)* | 8.3 (4.7) |
| Pain catastrophizing (PCS) (range: 0-52), mean (SD) | 10.8 (7.7) | 10.1 (7.5) | 14.9 (8.4)* | 8.9 (4.4) |
| Neuropathic like pain (PainDetect) (range: -1-38), mean (SD) | 9.5 (6.0) | 9.4 (6.2) | 10.4 (5.7) | 8.7 (5.2) |
| PPT OA joint (sex-standardized), mean (SD) † | 0.0 (1.0) | 0.0 (0.9) | -0.2 (1.3) | -0.0 (1.0) |
| PPT m. tibialis anterior (sex-standardized), mean (SD) † | 0.0 (1.0) | 0.0 (1.0) | 0.0 (1.2) | -0.2 (1.2) |
| Mechanical temporal summation (sex-standardized), mean (SD) † | 0.0 (1.0) | -0.0 (1.0) | 0.2 (1.1) | -0.1 (1.3) |

*Abbreviations*: ACR = American College of Rheumatology OA = osteoarthritis; SD = standard deviation; IQR = interquartile range; BMI = body mass index; ASES = arthritis self-efficacy scale; AUSCAN = Australian/Canadian Osteoarthritis Hand Index; KL = Kellgren-Lawrence; IMMPACT = Initiative on Methods, Measurement, and Pain Assessment in Clinical Trials; NRS = Numerical Rating Scale; HADS = Hospital Anxiety and Depression Scale; PCS = Pain Catastrophizing Scale; PPT= pain pressure threshold.

*=Differences with P<0.05.

†= standard deviation of sex-standardized values from QST: PPT OA: Women: 2.0 kilograms, men: 1.5 kilograms; PPT m. tibialis anterior: Women= 2.6 kilograms, men= 3.1 kilograms; Temporal summation: Women =1.6 NRS points, men= 1.3 NRS points

**Supplementary Table 6:**

Baseline indicator and clinical characteristics of participants transitioning (improved or worsened) versus those remaining in the same class throughout the follow-up period, results from the clinical model (N=213).

| **Measures** | **Sample (n=213)** | **Same class (n=168, 78.9%)** | **Improved (n=36, 16.9%)** | **Worsened (n=9, 4.2%)** |
| --- | --- | --- | --- | --- |
| ***Clinical characteristics*** |  |  |  |  |
| Age, mean (SD) years | 60.9 (6.0) | 60.6 (6.3) | 62.1 (4.8) | 60.8 (5.1) |
| No of women, n (%) | 185 (86.9) | 150 (89.3) | 28 (77.8) | 7 (77.8) |
| Higher education, n (%) | 130 (61.0) | 102 (60.7) | 21 (58.3) | 7 (78.8) |
| Working, n (%) | 124 (58.8) | 97 57.7) | 21 (58.3) | 6 (66.7) |
| Fulfils ACR hand criteria, n (%) | 202 (94.8) | 159 (94.6) | 34 (94.4) | 9 (100) |
| BMI, mean (SD) kg/m^2^ | 26.6 (4.8) | 26.6 (4.8) | 26.8 (5.1) | 25.0 (3.8) |
| Symptom duration (y), median (IQR) years | 6 (3-13) | 6 (2-13) | 6 (3-12) | 7 (3-17) |
| Comorbidity index sum score (range: 0-45), mean (SD) | 7.5 (4.1) | 7.3 (4.2) | 8.5 (4.0) | 7.6 (2.4) |
| ASES (range: 10-100), mean (SD) | 63.2 15.8) | 63.6 (15.9) | 60.9 (16.4) | 65.6 (13.0) |
| KL-sum score (range: 0-128), mean (SD) | 31.3 (18.9) | 31.7 (19.7) | 29.2 (15.6) | 32.6 (16.1) |
| Structure-pain concordance (%), mean (SD) | 60.9 (36.8) | 60.3 (38.1) | 63.1 (30.6) | 63.9 (37.7) |
| ***IMMPACT Indicators*** |  |  |  |  |
| NRS hand pain (range: 0-10), mean (SD) | 3.7 (2.1) | 3.6 (2.2) | 4.3 (2.2) | 2.4 (1.4) |
| NRS all bodily pain (range: 0-10), mean (SD) | 4.0 (2.3) | 3.7 (2.2) | 5.4 (2.3)* | 2.3 (0.9) |
| Sleep problems (15D) (range:1-4), mean SD | 2.25 (1.0) | 2.3 (1.0) | 2.2 (0.9) | 2.2 (1.0) |
| NRS fatigue, (range: 0-10), mean (SD) | 3.8 (2.8) | 3.6 (2.8) | 4.5 (3.0) | 4.2 (2.1) |
| Depression /Anxiety (HADS) (range: 0-42), mean (SD) | 6.9 (5.5) | 5.9 (5.2) | 11.0 (4.9)* | 8.2 (4.6) |
| Pain catastrophizing (PCS) (range: 0-52), mean (SD) | 10.8 (7.7) | 10.2 (7.6) | 14.2 (7.7)* | 8.4 (3.6) |
| Neuropathic like pain (PainDetect) (range: -1-38), mean (SD) | 9.5 (6.0) | 9.5 (6.2) | 10.1 (5.5) | 7.9 (5.5) |

*Abbreviations*: ACR = American College of Rheumatology OA = osteoarthritis; SD = standard deviation; IQR = interquartile range; BMI = body mass index; ASES = arthritis self-efficacy scale; AUSCAN = Australian/Canadian Osteoarthritis Hand Index; KL = Kellgren-Lawrence; IMMPACT = Initiative on Methods, Measurement, and Pain Assessment in Clinical Trials; NRS = Numerical Rating Scale; HADS = Hospital Anxiety and Depression Scale; PCS = Pain Catastrophizing Scale

*=Differences with P<0.05 versus those remaining in the same class.

**Supplementary table 7:** Syntaxes used for the latent transition analyses in R LMest.

library(LMest)

#load the data

load(file=”data.Rdata”)

#prepare the data

dta <- lmestData(data=data, id="ID",time="time", responsesFormula = Y1 + Y2 + Y3 + Y4 + Y5 + Y6 + Y9+ Y10 ~ NULL)

dta$data$ID = as.numeric(dta$id)

dta$data$time = as.numeric(dta$data$time)

#model with 2 to 6 classes

mod <- lmestCont(responsesFormula = Y1 + Y2 + Y3 + Y4 + Y5 + Y6 + Y9 + Y10 ~ NULL, index = c("ID","time"), data=dta$data, k=2:6, maxit=10000, tol = 10^-11,modBasic = 1, output = T, start=0)

plot(mod, what="modSel")
